# Assessment of perinatal anxiety: diagnostic accuracy of five measures

**DOI:** 10.1192/bjp.2023.174

**Published:** 2024-04

**Authors:** Susan Ayers, Rose Coates, Andrea Sinesi, Helen Cheyne, Margaret Maxwell, Catherine Best, Stacey McNicol, Louise R. Williams, Nazihah Uddin, Una Hutton, Grace Howard, Judy Shakespeare, James J. Walker, Fiona Alderdice, Julie Jomeen, the MAP Study Team

**Affiliations:** Centre for Maternal and Child Health Research, School of Health and Psychological Sciences, City University of London, UK; Nursing, Midwifery and Allied Health Professions Research Unit, University of Stirling, UK; Midwifery Department, King's College London, UK; Retired GP, Oxford, UK; Faculty of Medicine and Health, St James's University Hospital, University of Leeds, UK; National Perinatal Epidemiology Unit, Nuffield Department of Population Health, University of Oxford, UK; and School of Nursing and Midwifery, Queen's University Belfast, UK; Faculty of Health, Southern Cross University, Australia; MAP Study Team (see Acknowledgements)

**Keywords:** Anxiety or fear-related disorders, perinatal psychiatry, rating scales, diagnostic accuracy, depressive disorders

## Abstract

**Background:**

Anxiety in pregnancy and after giving birth (the perinatal period) is highly prevalent but under-recognised. Robust methods of assessing perinatal anxiety are essential for services to identify and treat women appropriately.

**Aims:**

To determine which assessment measures are most psychometrically robust and effective at identifying women with perinatal anxiety (primary objective) and depression (secondary objective).

**Method:**

We conducted a prospective longitudinal cohort study of 2243 women who completed five measures of anxiety and depression (Generalized Anxiety Disorder scale (GAD) two- and seven-item versions; Whooley questions; Clinical Outcomes in Routine Evaluation (CORE-10); and Stirling Antenatal Anxiety Scale (SAAS)) during pregnancy (15 weeks, 22 weeks and 31 weeks) and after birth (6 weeks). To assess diagnostic accuracy a sample of 403 participants completed modules of the Mini-International Neuropsychiatric Interview (MINI).

**Results:**

The best diagnostic accuracy for anxiety was shown by the CORE-10 and SAAS. The best diagnostic accuracy for depression was shown by the CORE-10, SAAS and Whooley questions, although the SAAS had lower specificity. The same cut-off scores for each measure were optimal for identifying anxiety or depression (SAAS ≥9; CORE-10 ≥9; Whooley ≥1). All measures were psychometrically robust, with good internal consistency, convergent validity and unidimensional factor structure.

**Conclusions:**

This study identified robust and effective methods of assessing perinatal anxiety and depression. We recommend using the CORE-10 or SAAS to assess perinatal anxiety and the CORE-10 or Whooley questions to assess depression. The GAD-2 and GAD-7 did not perform as well as other measures and optimal cut-offs were lower than currently recommended.

Mental health problems affect one in five women in pregnancy and the first year after giving birth (the perinatal period), with substantial impact on families and society.^1^ The total economic cost of perinatal mental health problems in the UK is estimated to be £8.1 billion for every annual cohort of births, with 72% of this cost attributable to the long-term effect on the child.^1^ The most common mental disorders are depression and anxiety, which often coexist. Although depression has been extensively researched, research on anxiety is critically needed. Perinatal anxiety affects an estimated 20% of women^2^ and is characterised by intense symptoms of anxiety and fear. Anxiety disorders include generalised anxiety disorder, panic, phobias, social anxiety, agoraphobia, obsessive–compulsive disorder and post-traumatic stress disorder.^3^ Evidence of the impact of perinatal anxiety on women and their infants includes increased risk of preterm birth, postnatal depression and poorer developmental outcomes for the infant.^4,5^ Evidence also shows that moderate symptoms which do not meet diagnostic thresholds can be distressing and debilitating.^6^

In most countries worldwide universal screening is not in place for mental health in the perinatal period. A few countries, such as the UK and USA, have clinical guidelines with varying recommendations for perinatal depression and anxiety screening and assessment.^7,8^ Anxiety is now recognised as being important to assess in itself and as a predictor of depression.^9^ In the UK, National Institute of Health and Care Excellence (NICE) guidelines^8^ suggest that health professionals ask two questions to identify anxiety at antenatal and postnatal appointments (the two-item Generalized Anxiety Disorder scale (GAD-2)^10^) and two questions to identify depression (the Whooley questions^11^). There is evidence that the Whooley questions may be useful as a generic measure to identify anxiety as well as depression.^12^ However, the measure recommended for anxiety has not been validated in perinatal populations so there is little evidence of its effectiveness in this population. Available research suggests that, although the GAD performs well at identifying generalised anxiety disorder, it is less sensitive to other anxiety disorders.^12^

Robust methods of assessing perinatal anxiety are essential if services are to identify and treat women and pregnant people with perinatal anxiety disorders or who have severe symptoms but do not meet clinical thresholds for a diagnosis of a disorder. Assessment methods need to be effective at discriminating between those who need intervention and those experiencing normal anxiety associated with pregnancy and birth. However, there is little evidence on the effectiveness of different methods of assessing perinatal anxiety. The overlap between anxiety and depression also needs to be considered, given that a general assessment may be more parsimonious and effective than separate assessments. The lack of evidence on perinatal anxiety assessment has an impact on policy and practice, with many clinical guidelines not recommending specific assessment tools. The need for research on this is recognised by clinical policy and research organisations, with the National Institute for Health and Care Research calling for rigorous evidence on methods of perinatal anxiety assessment that can be used by health and social care services to identify those in need of intervention.^13^ This study aimed to address this by evaluating five versions of four self-report assessment measures to determine which are the most psychometrically robust and effective (i.e. diagnostically accurate) at identifying women with perinatal anxiety (primary objective) or depression (secondary objective).

## Method

### Study design and population

**Fig. 1:**
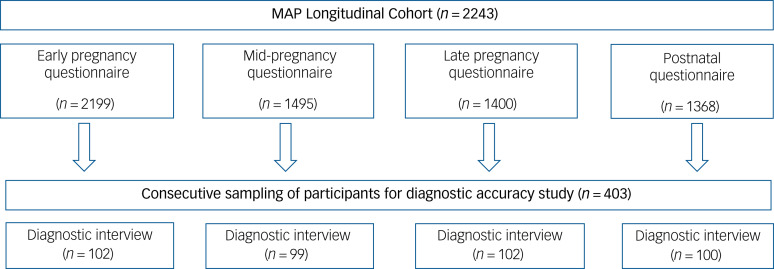
Sampling for the diagnostic accuracy study. MAP, Methods of Assessing Perinatal Anxiety study.

Methods of Assessing Perinatal Anxiety (MAP) was a longitudinal cohort study of 2243 women recruited from England and Scotland between November 2020 and November 2021. Results presented here are based on diagnostic interviews conducted with a subsample of 403 participants (Fig. 1). The study protocol (njl-admin.nihr.ac.uk/document/download/2034506) and registration details are available online (www.researchregistry.com/browse-the-registry#home/registrationdetails/5f50e17ebd7980001572b08e/).

Participants were recruited by research midwives/nurses or clinical midwives at 12 National Health Service (NHS) trusts in England and 5 NHS health boards in Scotland. Recruitment was done in-person or remotely around the time of the antenatal booking appointment or first pregnancy scan. Women were eligible for the MAP study if they were aged 16 years or over; less than 15 weeks pregnant at the time of recruitment; able to provide written informed consent; and had sufficient English to understand and complete questionnaires. Participants who were interested in taking part provided written informed consent. The research team then sent them self-report mental health assessment questionnaires to complete at three time points in pregnancy and one postnatally. These were early pregnancy (mean gestation 11.4 weeks, s.d. = 2.0, range 5–16); mid-pregnancy (mean gestation 23.0 weeks, s.d. = 1.3, range 21–27); late pregnancy (mean gestation 31.9 weeks, s.d. = 1.2, range 30–35); and postnatally (mean 7.9 weeks, s.d. = 2.4, range 4–17). Measures were completed online or by post according to the participants’ preferences. The order of presentation of the mental health assessment measures was counterbalanced to minimise risk of bias in response patterns.

Participants in the diagnostic accuracy sample (total *n* = 403: *n* = 352 in England, *n* = 51 in Scotland) were recruited as their questionnaire assessments were returned. Consecutive sampling was used to minimise bias, as recommended by guidelines for studies of diagnostic accuracy (QUADAS-2),^14^ and a 10:1 ratio of participants from England and Scotland was achieved, which reflects relative annual births for the two nations. Participants were contacted after their questionnaire was returned to request their participation in the diagnostic interview. If they consented a date for the diagnostic interview was arranged. Different participants were sampled at each time point (early pregnancy *n* = 102; mid-pregnancy *n* = 99; late pregnancy *n* = 102; postnatally *n* = 100) so that each participant was interviewed at only one time point. Once the required number of participants were interviewed for a particular time point recruitment stopped for this time point. Participants were assessed using a gold-standard diagnostic interview: the Mini-International Neuropsychiatric Interview version 7.0.2 (MINI).^15^ Modules of the MINI administered were Panic Disorder, Agoraphobia, Social Anxiety Disorder, Obsessive–Compulsive Disorder, Post-traumatic Stress Disorder, Generalised Anxiety Disorder, Specific Phobia and Major Depressive Episode (current and past).

Diagnostic interviews were conducted by three psychologists or other clinically qualified members of the research team, who were masked to results of the questionnaire assessment measures. Interviews were completed within 28 days of participants completing their questionnaire assessment. Interviews were conducted by telephone and audio-recorded to enable checks for fidelity and inter-rater reliability. Inter-rater reliability was examined for 5% of the interviews, which were coded by another member of the research team, and agreement was high (96%). Participants who met criteria for a current anxiety or depressive disorder were encouraged to see a health professional and given information about options such as helplines and self-referral to specialist services (*n* = 104). Safeguarding protocols were in place for participants who reported suicidal ideation (*n* = 1).

### Ethical approval

The authors assert that all procedures contributing to this work comply with the ethical standards of the relevant national and institutional committees on human experimentation and with the Helsinki Declaration of 1975, as revised in 2008. All procedures involving human participants were approved by the National Health Service West of Scotland Research Ethics Service (WoSREC 3), reference 20/WS/0065. Written informed consent was obtained from all participants.

### Choice of measures

Measures were identified on the basis of clinical utility (e.g. brevity, being used clinically) and research evidence that they were likely to be effective. Most of the measures are included in UK clinical guidance on perinatal outcome measures^8^ and care pathways for perinatal mental health.^16^ Five versions of four measures were included – three that assess perinatal anxiety and two that assess broader distress, as follows.

The GAD-7 consists of seven self-report items used to identify probable cases of GAD,^10^ according to criteria in DSM-IV.^17^ Items are scored on a 0–3 Likert scale and higher scores reflect greater anxiety severity. GAD-7 total scores range from 0–21, with a recommended cut-off score of 10.^10^

The GAD-2 is a two-item version of the GAD-7 and is the clinically recommended perinatal anxiety assessment in the UK.^8^ Evidence for using the GAD-2 with perinatal populations is mixed, with some studies finding poor diagnostic performance.^12^ GAD-2 total scores range from 0–6, with a recommended cut-off score of 3.^18^

The Clinical Outcomes in Routine Evaluation (CORE-10)^19^ is a ten-item measure of psychological distress derived from the larger CORE-OM measure. The ten items are scored on a 0–4 Likert scale. Total scores range from 0 to 40 and suggested cut-off scores are: 5–10, low-level problems; 10–15, mild psychological distress; 15–20, moderate distress; 20–25, moderately severe distress; and 25–40, severe psychological distress.^19^ One study suggests that the CORE-10 has good psychometric properties with pregnant populations and performs better than measures of anxiety (GAD-2) and depression (Whooley questions) at identifying those worried about their psychological health.^20^ The CORE-10 is recommended by the UK Royal College of Psychiatrists as a core outcome measure for perinatal services.^21^

The Stirling Antenatal Anxiety Scale (SAAS) is a ten-item, clinically derived measure developed specifically for perinatal anxiety. The SAAS includes general anxiety and pregnancy-specific anxiety items.^22^ The pregnancy-specific items are about the birth and baby so the scale can be used postnatally. Items are scored on a 0–4 Likert scale. Total scores range from 0 to 40 with a cut-off score of 8. The scale has good diagnostic accuracy and there is some evidence it performs better than the GAD-2 or GAD-7 at identifying women with perinatal anxiety.^22^

The Whooley questions are two yes/no questions widely used in maternity services to assess depression.^8^ Answering ‘yes’ to one or both of the questions indicates possible depression. There is evidence suggesting that the scale has high sensitivity, but variable specificity, in identifying perinatal depression,^23^ as well as limited evidence that it might also identify perinatal anxiety.^12^

Sample characteristics and information on obstetric and mental health history were recorded from self-reports in early pregnancy.

### Statistical analysis

Diagnostic accuracy was determined by comparing questionnaire assessments and diagnostic interviews in the same participants at the same time point. The true-positive rate (sensitivity), true-negative rate (specificity), positive and negative likelihood ratio values, negative predictive value and Youden's index score for each of the scales were calculated at a range of possible cut-off scores. A positive likelihood ratio >2 and a negative likelihood ratio <0.5 were considered acceptable, and a negative predictive value >0.8 and Youden's index score >0.5 were used as minimal values for the acceptability of cut-off scores.

Analyses of the area under the receiver operating curve (AUROC) were conducted to provide a single index of overall diagnostic performance. A value of 0.80 or above is considered acceptable for widespread application of a clinical screening tool. Examination of the AUROC for the five assessment measures enabled us to determine the appropriate cut-off scores for each of the measures.

Psychometric properties evaluated included internal consistency using Cronbach's alpha (α), where a value greater than 0.7 is considered acceptable. Item-total and inter-item correlations were also examined. Factor analysis was used to explore the factor structure of measures with more than two items (i.e. GAD-7, CORE-10, SAAS). Based on prior studies it was expected that scales would have one factor, or unidimensional structure, with an eigenvalue >1 used to determine how many factors should be retained. Sampling adequacy was assessed by the Kaiser–Meyer–Olkin (KMO) test, which indicted that the sample size was adequate for factor analysis (GAD-7: 0.88; CORE-10: 0.88; SAAS: 0.93).

Finally, as the five measures were examining similar constructs there should be positive correlations between the measures, demonstrating convergent validity. It was hypothesised there would be large correlations between the GAD-2, GAD-7, CORE-10 and SAAS measures, and a moderate correlation between these and the Whooley, given that it is a depression scale.

Analysis was conducted using Stata for Windows, version 16 or above. The analysis plan was pre-registered on the Open Science Framework (osf.io/yp2ae). Very few data were missing so information on this is given in the Supplementary materials available at https://dx.doi.org/10.1192/bjp.2023.174.

## Results

### Sample characteristics

The mean age of the sample was 34.2 years (s.d. = 4.6) (Table 1). The majority of participants were married (59.5%) or cohabitating (34.1%), educated to degree level or higher (71.9%) and White British (English, Welsh, Scottish, Northern Irish; 72.5%). Over half the sample (60.1%) had had a previous pregnancy, and just under 40% reported previous mental health problems, predominantly depression and anxiety. Across all time points the combined prevalence of anxiety disorders was 19.9% and depression was 6.0%.

**Table 1:** Sample characteristics (*n* = 403)

Variable	Total *n*^a^	*n*	%
Marital status	375		
In a relationship but not cohabitating		14	3.7
Cohabitating		128	34.1
Married/civil partnership		223	59.5
Separated/divorced/single		10	2.7
Education	377		
None		3	0.8
Secondary education		19	5.0
Post-secondary education		49	13.0
Vocational qualification		35	9.3
Undergraduate degree or equivalent		165	43.8
Postgraduate degree or equivalent		87	23.1
Doctorate		19	5.0
Ethnicity	378		
White (English/Welsh/Scottish/Northern Irish)		274	72.5
Black (African/Caribbean/Other)		13	3.4
Arab		3	0.8
Asian (Bangladeshi/Indian/Chinese/Pakistani/other)		31	8.2
Mixed/multiple ethnicity		13	3.5
Any other White background		44	11.6
Previous pregnancy	398	239	60.1
Previous miscarriage or stillbirth	398	124	31.2
Anxiety disorders (MINI)	403	80	19.9
Major depressive disorder (MINI)	400	24	6.0
Previous mental health disorder	394	152	38.6

MINI, Mini-International Neuropsychiatric Interview.

a. Missing values mean that total *n* range from 375 to 403.

### Diagnostic accuracy

The sensitivity, specificity, and positive and negative likelihood ratio values of the five scales at different cut-off scores are shown in Table 2. The GAD-2 showed good sensitivity and specificity using a cut-off score of 2, not the recommended cut-off score of 3,^8^ which had poor sensitivity (38%). The GAD-7's optimal cut-off score was also lower than specified in guidelines,^8^ with a cut-off score of 6 maximising both sensitivity and specificity (64.6% and 75.8% respectively). Again, although specificity was higher at the recommended cut-off score of 8 (88.2%), sensitivity suffered (45.5%).

**Table 2:** Sensitivity, specificity, positive likelihood ratio (LR+), negative likelihood ratio (LR−) and negative predictive values (NPV) for anxiety diagnosis

	Sensitivity, % (95% CI)	Specificity, % (95% CI)	LR+	LR−	NPV	Youden's index
GAD-2 cut-off scores						
**≥2**	**70.89 (66–75)**	**76.16 (72–80)**	**2.97**	**0.38**	**0.91**	**0.47**
≥3	37.97 (33–43)	92.57 (90–95)	5.11	0.67	0.86	0.31
≥4	29.11 (25–34)	95.98 (94–98)	7.23	0.74	0.85	0.25
GAD-7 cut-off scores						
**≥6**	**64.56 (60–69)**	**75.78 (72–80)**	**2.67**	**0.47**	**0.90**	**0.40**
≥7	55.70 (51–61)	82.92 (79–87)	3.26	0.53	0.88	0.39
≥8	45.57 (41–50)	88.20 (85–91)	3.86	0.62	0.87	0.34
≥9	41.77 (37–47)	90.68 (88–94)	4.48	0.64	0.86	0.32
CORE-10 cut-off scores						
**≥9**	**69.62 (65–74)**	**78.95 (75–83)**	**3.31**	**0.38**	**0.91**	**0.49**
≥10	64.56 (60–69)	82.35 (79–86)	3.66	0.43	0.90	0.47
≥11	59.49 (55–64)	86.69 (83–90)	4.47	0.47	0.90	0.46
≥12	56.96 (52–62)	88.85 (86–92)	5.11	0.48	0.89	0.46
SAAS cut-off scores						
**≥9**	**83.54 (80–87)**	**72.76 (68–77)**	**3.07**	**0.23**	**0.95**	**0.56**
≥10	77.22 (73–81)	74.61 (70–79)	3.04	0.31	0.93	0.52
≥11	68.35 (64–73)	76.16 (72–80)	2.87	0.42	0.91	0.44
≥12	65.82 (61–70)	79.26 (75–83)	3.17	0.43	0.90	0.45
Whooley cut-off scores						
**≥1**	**58.75 (54–64)**	**75.54 (71–80)**	**2.40**	**0.55**	**0.88**	**0.34**
≥2	42.50 (38–57)	92.88 (90–95)	5.97	0.62	0.87	0.35

GAD-2, two-item Generalized Anxiety Disorder scale; GAD-7, seven-item Generalized Anxiety Disorder scale; CORE-10, ten-item Clinical Outcomes in Routine Evaluation; SAAS, Stirling Antenatal Anxiety Scale; Whooley, Whooley questions.

Values in bold indicate optimal cut-off scores.

The CORE-10 showed good sensitivity (64.6%) and excellent specificity (82.3%) at a cut-off score of 9. The SAAS showed excellent sensitivity (83.5%) and very good specificity (72.7%) at a cut-off score of 9. As per clinical guidelines, a cut-off score of 1 for the Whooley was optimal, with good sensitivity (58.7%) and very good specificity (75.5%).^8^ Increasing the cut-off score on the Whooley to 2 increased specificity to 92.9% but sensitivity suffered (42.5%).

**Fig. 2:**
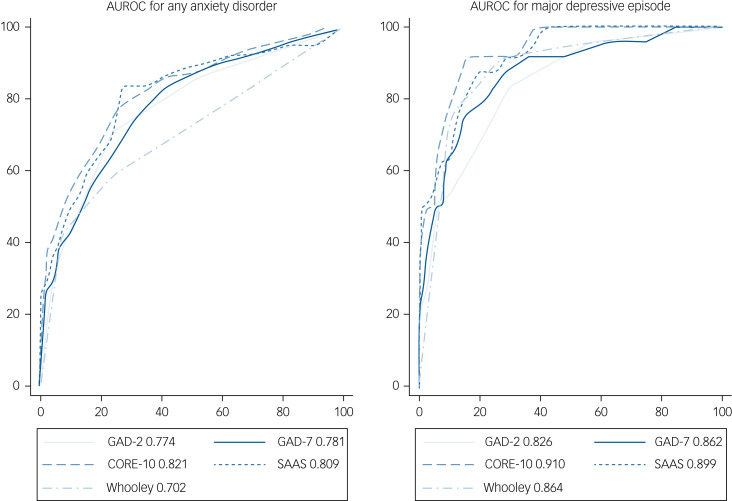
Area under receiver operating curve (AUROC) for all measures for diagnosis of any anxiety disorder and of major depressive disorder. GAD-2, two-item Generalized Anxiety Disorder scale; GAD-7, seven-item Generalized Anxiety Disorder scale; CORE-10, ten-item Clinical Outcomes in Routine Evaluation; SAAS, Stirling Antenatal Anxiety Scale; Whooley, Whooley questions.

Figure 2 shows AUROC curves for anxiety and depression for each of the scales. For anxiety, the CORE-10 and SAAS had good discriminative ability (CORE-10: 0.82, 95% CI 0.77–0.88; SAAS: 0.81, 95% CI 0.75–0.87) but the other scales had discriminative ability below the acceptable threshold. The Whooley had the lowest AUROC value (0.70, 95% CI 0.64–0.77) followed by the GAD-2 (0.77, 95% CI 0.72–0.83) and GAD-7 (0.78, 95% CI 0.72–0.84).

These findings suggest that the CORE-10 and SAAS were the most accurate diagnostic measures. At the optimal cut-off scores identified, the SAAS had the highest sensitivity (probability of a questionnaire score indicating anxiety in someone who does have anxiety) and the CORE-10 had the highest specificity (probability of a questionnaire score indicating no anxiety in someone who does not have anxiety).

For depression, the CORE-10, SAAS, GAD-7 and Whooley all had acceptable diagnostic accuracy. The optimal cut-off score on each measure was the same for depression as it was for anxiety diagnoses, with all measures reaching the thresholds of a positive likelihood ratio greater than 2, negative likelihood ratio less than 0.5, a negative predictive value greater than 0.8 and a Youden's index greater than 0.5 for a diagnosis of depression. For depression, the highest sensitivity and specificity were shown by the CORE-10 (91.67% sensitivity, 73.28% specificity) and Whooley (91.67% sensitivity, 72.56% specificity). The SAAS had the same sensitivity but lower specificity (65.08%) (see Supplementary materials).

### Psychometric properties

Internal reliability was good for the CORE-10 (α = 0.84), GAD-7 (α = 0.89) and SAAS (α = 0.91). It was not calculated for the GAD-2 or Whooley questions because they each include only two items so Cronbach's test was not appropriate. Item-total correlations showed that all measures were in the range 0.54–0.93. Inter-item correlations revealed a range of moderate to moderately high inter-item correlations, which is desirable for items in a scale, with correlations all above 0.20 and below 0.80 (range 0.25–0.77). Inspection of response distributions showed no floor or ceiling effects for items in the GAD-2, GAD-7, SAAS or Whooley scales. However, there was a floor effect for an item in the CORE-10, where all participants answered ‘not at all’ to the item ‘I have made plans to end my life’.

Results of factor analysis showed that all three measures had a unidimensional structure as expected, with only one factor having an eigenvalue >1 (see Supplementary materials). This suggests that all items on each scale are measuring the same latent construct.

Convergent and discriminant validity were good (see Supplementary materials). Significant positive correlations were found between all measures, with coefficients suggesting strong positive relationships (coefficient range 0.50–0.86), indicating good convergent validity. As expected, the smallest coefficients were between the Whooley and other scales (range 0.50–0.54) because it is a measure of depression.

## Discussion

This study evaluated five assessment measures to determine whether they are psychometrically robust and effective at identifying perinatal anxiety (primary objective) or depression (secondary objective). All measures performed well identifying anxiety or depressive disorders, based on the MINI interview. The best diagnostic accuracy for anxiety was observed in the CORE-10 and SAAS. The best diagnostic accuracy for depression was observed in the CORE-10 and Whooley questions. Optimal cut-off scores for each measure were the same for identifying anxiety or depression and measures were psychometrically robust.

This research has a number of implications. Importantly, results support the use of self-report measures in universal assessment of perinatal anxiety or depression, as most measures met criteria for good or excellent diagnostic accuracy. This did not include the GAD and Whooley questions, which only had ‘fair’ discriminative ability for anxiety. Optimal cut-offs on the GAD were also lower than recommended in clinical guidelines, which suggests that perinatal anxiety might differ from anxiety in the general population.^8^ The poor performance of the GAD is consistent with research showing its poor diagnostic accuracy for perinatal anxiety.^12^

Interestingly, all the measures had good or excellent diagnostic accuracy for depression, despite the fact that most were developed to measure different constructs (e.g. the SAAS was developed to measure perinatal anxiety) and measures were chosen on the basis of probable performance for assessing anxiety. Two measures performed well at identifying both anxiety and depression: the CORE-10 and SAAS. This may be because symptoms of anxiety and depression are overlapping, meaning that distinguishing between them is difficult or even unnecessary given their substantial comorbidity. This is consistent with theories arguing that anxiety and depression are not separate disorders but variations on axes of mood.^24^ It is also possible that good scale construction makes measures sensitive to a range of affective symptoms. Indeed, the CORE-10 was developed to measure various symptoms of distress and this study confirms that it is accurate at identifying perinatal anxiety and depression. In contrast, the SAAS was developed to measure pregnancy-related anxiety so it is surprising that it has good diagnostic accuracy for depression, although it had lower specificity for depression than the CORE-10 or Whooley questions, consistent with its focus on anxiety.

This research can inform clinical guidelines on assessment of perinatal anxiety. When assessing perinatal anxiety, results suggest that the CORE-10 or SAAS are most effective. As a secondary objective, results suggest that the CORE-10 or Whooley questions are effective measures of depression, although we did not include many measures of depression so measures not included here may also be effective, such as the Edinburgh Postnatal Depression Scale. Results suggest optimal cut-offs of 9 or more on the SAAS for perinatal anxiety disorders, 9 or more on the CORE-10 for perinatal anxiety or depressive disorders and 1 or more on the Whooley for perinatal depressive disorder.

Which measure health and social care services choose to use will partly depend on how the measure is utilised. For example, if a service wants to assess for anxiety and depression separately they might employ the SAAS and Whooley, or SAAS and CORE-10 respectively. If a service prefers a one-off screening tool to identify women with anxiety and/or depression they might employ the CORE-10 and then follow-up women who score over the cut-off scores with a more detailed assessment. Decisions about which approach and measure to use will be influenced by current practice, service constraints, characteristics and preferences with regard to the length of the scale, ease of use, patient burden, etc.

Decisions about assessment also need to consider the balance between sensitivity and specificity at different points in the care pathway. For initial assessment it is important that a measure has high sensitivity (i.e. picks up most women who have potential anxiety) to minimise false negatives, which might result in those with anxiety being missed. Once those at risk of anxiety disorders are identified, subsequent assessment might prioritise specificity (i.e. identifying people with the disorder) to minimise false positives, which might result in those without a disorder being referred to specialist services.

### Strengths and limitations

This study is the first to establish the diagnostic accuracy and effectiveness of these measures in a large non-clinical perinatal population. However, participants were highly educated compared with the UK population, and a large proportion reported previous mental health problems. No participants in this sample reported suicidal ideation in the questionnaires, although one participant reported it in the diagnostic interview (and it was reported by a few participants in the larger cohort). This research would therefore benefit from replication in different samples. We examined five measures but it is possible that other measures may perform as well or better than those tested, particularly for depression. We focused on brief self-report measures as these are more likely to be adopted in busy, routine clinical practice. Our recommendations are therefore that the recommended measures are likely to be effective, but not necessarily superior to measures not included.

In conclusion, this study identified robust and effective methods of assessing perinatal anxiety. Recommendations are that the CORE-10 or SAAS are used for assessment and identification of perinatal anxiety. Results also suggest the CORE-10 or Whooley are effective for assessment and identification of perinatal depression. The GAD-7 and GAD-2 did not perform well and optimal cut-offs were lower than currently used in clinical practice, so these measures are not recommended. Further research is needed in more diverse samples, and to examine diagnostic accuracy for depressive and other disorders.

## Supporting information

Ayers et al. supplementary material Ayers et al. supplementary material

## Data Availability

Individual participant-level data are not available but research material, analytic codes and sample-level data and information are available from the corresponding author, on reasonable request.
