# Supplementary material for: Assessment of perinatal anxiety: diagnostic accuracy of five measures
Source: Br J Psychiatry. 2024 Apr;224(4):132–8. doi: 10.1192/bjp.2023.174 (PMC10933560; doi:10.1192/bjp.2023.174)
Supplement: Ayers et al. supplementary material [file S0007125023001745sup001.docx]

# Supplementary Materials

## Missing data

For the GAD-2/7, CORE-10, and SAAS one participant had missing data on all items and was excluded from analyses related to those variables. Another participant had one item missing on the GAD-7, CORE-10, and SAAS. As this was only 1 item, and less than 5% of the total items, this was replaced with the median value for that item. There were no missing data for the Whooley.

For the demographic variables there were some missing data: 19 (4.7%) had missing data regarding their age, 26 (6.45%) for their education level, 25 (6.2%) for their ethnicity, 24 for their marital status (6.95%), 10 (2.48%) on whether they had a previous pregnancy, and 30 (7.44%) on whether they had a previous mental health problem. There was no significant association between having missing data and being diagnosed through the MINI interviews with an anxiety disorder (all p>.05).

## Diagnostic accuracy of measures for a diagnosis of depression

**Table S1.** Sensitivity, specificity, positive LR, negative LR and negative predictive value for a diagnosis of major depressive disorder

| GAD-2 cut-points | Sensitivity (95% CI) | Specificity (95% CI) | LR+ | LR- | NPV | Youden’s Index |
| --- | --- | --- | --- | --- | --- | --- |
| **≥ 2** | **83**⋅**33% (80%-87%)** | **70**⋅**11% (66%-75%)** | **2**⋅**79** | **0**⋅**24** | **0**⋅**99** | **0**⋅**53** |
| ≥ 3 | 54⋅17% (49%-59%) | 89⋅15% (86%-92%) | 4⋅99 | 0⋅51 | 0⋅97 | 0⋅43 |
| ≥ 4 | 50% (45%-55%_ | 93⋅65% (91%-96%) | 7⋅88 | 0⋅53 | 0⋅97 | 0⋅44 |
| GAD-7 cut-points | |  |  |  |  |  |
| **≥ 6** | **87**⋅**5% (84%-91%)** | **71**⋅**43% (67%-76%)** | **3**⋅**06** | **0**⋅**18** | **0**⋅**99** | **0**⋅**59** |
| ≥ 7 | 79⋅17% (75%-83%) | 78⋅84% (75%-83%) | 3⋅74 | 0⋅26 | 0⋅98 | 0⋅58 |
| ≥ 8 | 75% (71%-79%) | 85⋅19% (82%-89%) | 5⋅06 | 0⋅29 | 0⋅98 | 0⋅60 |
| CORE-10 cut-points | |  |  |  |  |  |
| **≥ 9** | **91**⋅**67% (89%-94%)** | **73**⋅**28% (69%-78%)** | **3**⋅**43** | **0**⋅**11** | **0**⋅**99** | **0**⋅**65** |
| ≥ 10 | 91⋅67% (89%-94%) | 77⋅25% (73%-81%) | 4⋅03 | 0⋅11 | 0⋅99 | 0⋅74 |
| ≥ 11 | 91⋅67% (89%-94%) | 82⋅01% (78%-86%) | 5⋅10 | 0⋅10 | 0⋅99 | 0⋅74 |
| ≥ 12 | 91⋅67% (89%-94%) | 84⋅39% (81%-88%) | 5⋅87 | 0⋅10 | 0⋅99 | 0⋅76 |
| SAAS cut-points | |  |  |  |  |  |
| **≥ 9** | **91**⋅**67% (89%-94%)** | **65**⋅**08% (60%-70%)** | **2**⋅**63** | **0**⋅**13** | **0**⋅**99** | **0**⋅**57** |
| ≥ 10 | 91⋅67% (89%-94%) | 67⋅99% (63%-73%) | 2⋅86 | 0⋅12 | 0⋅99 | 0⋅6 |
| ≥ 11 | 91⋅67% (89%-94%) | 71⋅16% (67%-76%) | 3⋅18 | 0⋅12 | 0⋅99 | 0⋅63 |
| ≥ 12 | 87⋅5% (84%-91%) | 74⋅07% (70%-78%) | 3⋅38 | 0⋅17 | 0⋅99 | 0⋅62 |
| Whooley cut-points | |  |  |  |  |  |
| **≥ 1** | **91**⋅**67% (89%-94%)** | **72**⋅**56% (68%-77%)** | **3**⋅**34** | **0**⋅**11** | **0**⋅**99** | **0**⋅**64** |
| ≥ 2 | 75% (71%-79%) | 89⋅71% (87%-93%) | 7⋅29 | 0⋅28 | 0⋅98 | 0⋅65 |

**Table S2:** Eigenvalues from Factor Analysis on the GAD-7, CORE-10 and SAAS measures

| Factors | Eigenvalue | % of variance | Cumulative % |
| --- | --- | --- | --- |
| GAD-7 | | | |
| Factor1 | 3⋅75 | 37⋅5% |  |
| Factor2 | 0⋅30 | 3⋅0% | 40⋅5% |
| CORE-10 | | | |
| Factor1 | 3⋅72 | 37⋅2% |  |
| Factor2 | 0⋅37 | 3⋅7% | 40⋅9% |
| SAAS | | | |
| Factor1 | 5⋅26 | 52⋅6% |  |
| Factor2 | 0⋅45 | 4⋅5% | 57⋅1% |

Only factors with eigenvalues above ⋅30 are reported.

**Table S3.** Correlation matrix showing correlations between scales

|  | GAD-2 | GAD-7 | CORE-10 | SAAS |
| --- | --- | --- | --- | --- |
| GAD-2 |  |  |  |  |
| GAD-7 | 0⋅86* |  |  |  |
| CORE-10 | 0⋅70* | 0⋅76* |  |  |
| SAAS | 0⋅78* | 0⋅82* | 0⋅74* |  |
| Whooley | 0⋅50* | 0⋅52* | 0⋅54* | 0⋅53* |

* Indicates significance at ⋅05 level.
